# Exploring the standardized detection and sampling methods of human nasal SARS-CoV-2 RBD IgA

**DOI:** 10.3389/fimmu.2025.1571418

**Published:** 2025-05-20

**Authors:** Xuanxuan Zhang, Jizong Jia, Chaoying Hu, Yulong Fu, Guanxing Liu, Yajing Li, Qian He, Fan Gao, Na Li, Lina Wang, Jianping Chu, Henggang Xu, Zhihao Fu, Hui Zhao, Zhenglun Liang, Jingxin Li, Miao Xu, Qunying Mao

**Affiliations:** ^1^ State Key Laboratory of Drug Regulatory Science, Evaluation of Biological Products, Key Laboratory of Research on Quality and Standardization of Biotech Products, Institute of Biological Products, National Institutes for Food and Drug Control, Beijing, China; ^2^ Research Units of Innovative Vaccine Quality Evaluation and Standardization, Chinese Academy of Medical Sciences, Beijing, China; ^3^ Beijing Wantai Biological Pharmacy Enterprise Co., Ltd, Beijing, China; ^4^ School of Life Science and Biopharmaceutics, Shenyang Pharmaceutical University, Shenyang, China; ^5^ Changchun Institute of Biological Products Co., Ltd., Changchun, China; ^6^ Shanghai Institute of Biological Products Co., Ltd., Shanghai, China; ^7^ Beijing Minhai Biotechnology Co., Ltd., Beijing, China; ^8^ Jiangsu Provincial Centre for Disease Control and Prevention, Public Health Research Institute of Jiangsu Province, Nanjing, Jiangsu, China

**Keywords:** nasal antibody, nasal sample collection, binding activity, immune assay, SARS-CoV-2

## Abstract

**Introduction:**

Vaccines capable of effectively inducing mucosal immunity, particularly specific IgA antibodies, represent an ideal strategy for preventing infections and the transmission of pathogens such as SARS-CoV-2 and influenza viruses that rapidly replicate in the upper respiratory tract and cause clinical symptoms. However, a lack of standardized nasal antibody detection and sampling methods has hindered cross-study comparability and vaccine development.

**Methods:**

This study uses SARS-CoV-2 as a model pathogen to standardize nasal antibody detection methods and sampling methods. Following the scientific guidelines (Q14 and Q2(R2)) for analytical procedure development and validation released by the International Council for Harmonization (ICH), an ELISA for nasal SARS-CoV-2 WT-RBD specific IgA detection was established and validated. To compare the sampling methods, nasal samples were collected from five groups using three commonly used nasal sampling methods (M1: nasopharyngeal swab; M2: nasal swab; M3: expanding sponge method). The total IgA and SARS-CoV-2 WT-RBD IgA in clinical samples were detected.

**Results:**

The first validated ELISA for nasal SARS-CoV-2 WT-RBD specific IgA detection was established through analytical target profiling (ATP), risk assessment, and design of experiment optimization. Systematic validation demonstrated exclusive specificity for the target antigen, with intermediate precision of <17% and relative bias of <±4%, meeting ATP requirements. Analysis of 154 clinical samples demonstrated strong concordance between the novel method and electrochemiluminescence assays, with a concordance correlation coefficient of 0.87 for quantitative results and a kappa coefficient of 0.85 for results above and below the dilution-adjusted limit of quantification (LOQ). Applying this novel method, a clinical comparison revealed that M3 achieved superior performance in terms of the single-day detection rate (above dilution-adjusted LOQ 95.5%), 5-day consecutive detection rate (above dilution-adjusted LOQ 88.9%), and median SARS-CoV-2 WT-RBD IgA concentration (171.2 U/mL), significantly outperforming M1 (68.8%; 48.7%; 28.7 U/mL, p<0.0001) and M2 (88.3%; 77.3%; 93.7 U/mL, p<0.05).

**Conclusion:**

This study has established the first standardized nasal detection system. The system can be adapted with appropriate modifications for the clinical evaluation of other respiratory mucosal vaccines, thereby advancing the development of mucosal vaccines.

## 1 Introduction

Respiratory viruses, such as influenza, SARS-CoV-2, and RSV, exhibit a “hit-and-run” infection mode, characterized by rapid replication in respiratory epithelial cells and transmission to new hosts before the host’s adaptive immune response becomes effective (1). These viruses pose a significant threat to global public health (2–4). Although multiple intramuscular vaccines against influenza and SARS-CoV-2 have been approved or authorized for emergency use, they demonstrate limited efficacy in preventing infection despite reducing the risks of severe illness, hospitalization, and death. Over the past 20 influenza seasons, the effectiveness of influenza vaccines ranged at 14–60% (5). Notably, the fourth dose of prototype SARS-CoV-2 mRNA vaccines showed only 11–30% efficacy against symptomatic Omicron infection one month post-administration (6). This limitation primarily stems from the inability of intramuscular vaccines to induce mucosal immune responses in the upper respiratory tract, particularly antigen-specific IgA antibody responses, which are critical for blocking viral entry, replication, and transmission in mucosal epithelial cells during early infection (7, 8). In contrast, natural infection and intranasal vaccination can effectively elicit the production of nasal SARS-CoV-2-specific IgA (9–11).

IgA is the dominant antibody on mucosal surfaces, which are crucial for blocking viral entry, replication, and transmission in mucosal epithelial cells during the early stages of infection. (9). Compared to serum IgG and IgA, nasal SARS-CoV-2-specific IgA exhibits superior binding affinity, neutralizing capacity, and efficiency in inhibiting spike protein-mediated cell fusion, thereby preventing viral spread within the mucosal epithelia (9, 12). Additionally, a correlation has been found between nasal antigen-specific IgA levels and protection against infection (13–15). Consequently, developing vaccines capable of eliciting antigen-specific mucosal IgA responses is considered crucial for preventing “hit-and-run” respiratory viruses, such as SARS-CoV-2 and influenza (16).

Immunogenicity is a critical metric for evaluating vaccine efficacy. Serum antibodies are the primary endpoint for most intramuscular vaccines. However, for mucosal vaccines, relying solely on serum antibodies overlooks the immune responses elicited at mucosal sites (17). Currently, no standardized method exists for detecting nasal mucosal IgA. Some studies have employed high-sensitivity, high-throughput electrochemiluminescence (ECL) assays (14, 18, 19); however, these assays were originally developed for serum IgA detection. In addition, the specialized equipment required limits their widespread adoption. Alternative studies have used in-house ELISA methods; however, their sensitivity in clinical applications remains unverified.

Unlike uniformly distributed and easily accessible serum IgA, mucosal IgA forms a “flypaper-like” layer over mucosal surfaces, with substantial concentration variations across anatomical sites, making sample collection challenging (18, 20–25). Current nasal sampling methods, including washing, swabbing, and adsorption, vary widely with reported collection capability differences of up to 5-fold (26). The absence of standardized nasal IgA detection assays and sampling methodologies severely compromises cross-study comparability, hinders research on mucosal immune correlates of protection, and delays the development and application of mucosal vaccines (27, 28).

In our study, the concentration disparity between nasal mucosal and serum antibodies was quantified first. Two critical bottlenecks, sampling and detection methodologies, were addressed with the target of SARS-CoV-2 RBD-specific IgA. Standardized sampling and detection methodologies can accelerate research on the mucosal immune correlates of protection as well as advance the development of mucosal vaccines.

## 2 Methods

### 2.1 Study design and participants

The aim of this study was to compare the collection capabilities of three commonly used nasal sampling methods and to investigate the levels of SARS-CoV-2 WT-RBD IgA in the nasal mucosa across different populations.

Eligible subjects were individuals aged 18 years or older. Individuals eligible for inclusion were those who exhibited no symptoms of COVID-19 since the COVID-19 pandemic, or had a history of SARS-CoV-2 infection (with the last infection occurring 0.5–3 months or more than 10 months prior to sampling), or had received the SARS-CoV-2 mucosal vaccine within the past 3 months. Prior SARS-CoV-2 infection was self-reported by participants and defined as either a positive antigen rapid test or nucleic acid test result, or the acute onset of at least two typical symptoms or signs of COVID-19 with documented exposure to probable or confirmed cases, in the absence of etiological testing. Consecutive sampling was carried out with daily health checks to avoid the influence of participants’ health changes on the results. Individuals exhibiting suspected COVID-19 or influenza symptoms during 5 consecutive sampling days were excluded. The population could be stratified into five groups based on infection status and the type of mucosal vaccine administered (A: individuals without COVID-19 infection symptoms since the COVID-19 pandemic; B: individuals with the last SARS-CoV-2 infection occurring 0.5–3 months prior to sampling; C: individuals with the last SARS-CoV-2 infection occurring more than 10 months prior to sampling; D: individuals had a history of SARS-CoV-2 infection and intranasally vaccinated with single dose of live-attenuated influenza virus vector-based SARS-CoV-2 vaccine within the past 3 months; E: individuals had a history of SARS-CoV-2 infection and vaccinated with single dose of Ad5-nCoV vaccine through oral inhalation within the past 3 months). Each group was expected to recruit 33 participants. In fact, only Groups B and C successfully recruited the full quota of 33 participants, whereas Groups A, D, and E recruited 26, 30, and 32 participants, respectively (**Supplementary Table S1**).

Nasal samples were collected from all participants using three commonly used nasal collection methods (nasopharyngeal swab, nasal swab, expanding sponge method) for 5 consecutive days. Additionally, serum samples were collected from all participants.

### 2.2 Collection of nasal mucosal lining fluids

Nasopharyngeal swab (M1): A nylon flocked swab (Copan Diagnostics, Murrieta, CA, USA) was inserted into the left nostril to the nasopharyngeal region, rotated once, and stayed in the nasopharyngeal region for 15 seconds. Nasal swab (M2): A cotton swab (cat no: LKY-X-X2, Likangyuan medical device, Beijing, China) was inserted into the left nostril approximately 2 cm from the level of the nasal turbinate and rotated 30 times. Expanding sponge method (M3): A polyvinyl alcohol sponge (cat no.: PVF-J, Beijing Yingjia Medic Medical Materials Co., Ltd, China) was soaked in 50 mL of physiological saline to expand, placed into a 10 mL disposable syringe, and the plunger was pushed to the 4 mL mark to expel the fluid. Using sterile scissors, the dehydrated sponge was divided into two equal parts, and each part was cut into three equal pieces. One piece was inserted into the right nostril and left in place for 5 min. Samples collected by the three methods were each placed into a 1.5 mL UTM universal transport medium (Copan Diagnostics). Within 4 h of sampling, the swabs were removed, or the sponge’s absorbed liquid was expelled using a syringe, followed by centrifugation (room temperature, 1000 rpm, 3 min) and aliquoting.

### 2.3 Serum specimen collection

Blood samples were obtained from participants using BD vacutainer evacuated blood collection tubes. The samples were clotted for at least 30 min at room temperature and centrifuged at 2000 × *g* for 10 min. The supernatant was collected, aliquoted, and stored at −80°C.

### 2.4 Detection of immunoglobulin in nasal and serum samples

According to the instructions of the Human/NHP Kit (cat no. K15203D, Meso Scale Diagnostics, Rockville, MD, USA), human nasal swabs from Group B numbered 1–20 were diluted 10,000-fold, and paired serum samples were diluted 250,000-fold for the detection of IgA, IgG, and IgM. To assess the collection capability of three commonly used methods for retrieving total IgA, the Human/NHP kit was employed to analyze all mucosal samples collected via methods M1, M2, and M3. Each mucosal sample was diluted 10,000-fold prior to analysis.

### 2.5 Screening of anti-IgA specific antibodies

Human serum samples were collected as raw materials for preparation, and after primary purification by PEG precipitation, they were further purified by two-step chromatography columns using Protein G and Protein L. The purified human IgA protein, with a purity of 90%, was used as the immunogen to immunize mice. Two weeks after the third immunization, blood samples were collected, and antibody titers were measured using an indirect ELISA. Spleen cells from mice with serum titers of around one million were selected for cell fusion with myeloma cells. After fusion, culture supernatants were collected and tested using the indirect ELISA method. The absorbance at OD450/630 was measured using a microplate reader. A cutoff of OD > 0.2 was used to determine positivity. Based on cell growth in the culture plates and positivity results, six positive hybridoma cell lines were selected for subsequent experiments. The anti-IgA antibodies produced by these six cells were designated as W1-6. After large-scale production of W1-6, each antibody was diluted to a concentration of 10 μg/mL and subjected to eight 5-fold serial dilutions. Their binding capacities to purified IgA were then assessed using an indirect ELISA.

The hybridoma cell line (monoclonal cell line number: 5F5A4F3, deposit number: CGMCC No. 46012) of the mouse anti-human immunoglobulin A monoclonal antibody W2 was deposited at the General Microbiology Center of the China General Microbiological Culture Collection Center.

### 2.6 Dot blot assay

The dot blot assay was employed to evaluate the specificity of the three commercial enzyme-labeled antibodies (1: goat anti-human IgA (α-chain specific)-HRP, cat no. A0295, Sigma, GER; 2: mice anti-human IgA, cat no. A0053, Wuhan Aoke Botai Biotechnology Co., Ltd, China; 3: rabbit anti-human IgA, cat no. ab193189, Abcam, UK) and W2 against different types of human immunoglobulins. Human IgA, IgM, and IgG purified from serum with a purity of 90% were diluted to 50 μg/mL and then subjected to a 4-fold serial dilution across five gradients. A 2 μL aliquot of each dilution was spotted onto a PVDF membrane. Membranes were blocked with 5% skim milk. Enzyme-labeled antibodies were diluted 1:1000 in 5% skim milk and incubated with the membrane. Four PVDF membranes were transferred into these working solutions and incubated at 37°C for 1 h. After three additional washes with PBS containing 0.05% Tween 20, color development was performed using AEC substrate (cat no.: A2010, Solarbio, China).

### 2.7 Method development

#### 2.7.1 Establishment of the analytical target profile

In accordance with the ICH Q14 and Q2(R2) guidelines, a rapid, simple, quantitative, highly sensitive, and specific method for detecting SARS-CoV-2 mucosal IgA was developed. ATP was defined based on the method’s intended use, prior knowledge, and required performance characteristics (e.g., specificity, precision, and accuracy). Detection principles and acceptance criteria for specificity, precision, and accuracy were also established (**Table 1**).

**Table 1:** Comparison of ATP and final development results.

	Objectives	Results
Intended purpose	Detection of nasal SARS-CoV-2 RBD IgA antibodies in clinical samples	
Link to critical quality attribute	The RBD protein of SARS-CoV-2 is pre-coated in microplate wells. Nasal samples are added, and SARS-CoV-2 RBD IgA antibodies in the samples form a “coated antigen-antibody” complex with the coated antigen. Non-binding substances are washed away, and enzyme-labeled antibodies (monoclonal antibodies against human IgA labeled with horseradish peroxidase) are added for incubation, forming a “coated antigen-antibody-enzyme-labeled antibody” complex. After washing again, TMB substrate is added, and the HRP on the complex catalyzes the color reaction. If SARS-CoV-2-specific IgA antibodies are present in the sample, a blue product is produced, which turns yellow after termination; if SARS-CoV-2-specific IgA antibodies are absent, no color change occurs. The OD value after termination is detected by a microplate reader, and the content of SARS-CoV-2-specific IgA antibodies is calculated using a standard curve.	
Specificity	No cross-reactivity with rhinovirus, influenza A, influenza B, human metapneumovirus, and negative mucosal samples	No cross-reactivity
Intermediate Precision	≤20%	<17%
Accuracy (Relative bias)	≤±20%	<±4%

#### 2.7.2 Identification of critical impact factors

Factors influencing ELISA performance were analyzed and visualized using a fishbone diagram. A risk assessment was conducted to evaluate the impact of each factor on accuracy, precision, and specificity. Critical factors were prioritized for further optimization via the Design of Experiments (DoE) (**Supplementary Table S2**).

#### 2.7.3 Optimization of critical factors

##### 2.7.3.1 Screening of antigen coating concentration and enzyme-labeled secondary antibody dilution

WT RBD protein expressed in 293F from the codon-optimized RBD sequence of SARS-CoV-2 spike protein (GenBank accession number MN908947) was diluted to four concentrations (4 μg/mL, 2 μg/mL, 1 μg/mL, 0.5 μg/mL) for microplate coating. Enzyme-labeled antibodies were tested at four concentrations (2 μg/mL, 1 μg/mL, 0.5 μg/mL, and 0.25 μg/mL). Checkerboard titration was performed using the National Standard for anti-SARS-CoV-2 mucosal IgA (No. 300052-202401, 1000 U/mL) at 50 U/mL to determine the optimal conditions (29).

##### 2.7.3.2 Optimization of reaction time

The Method Operable Design Region (MODR) was analyzed to optimize the incubation times for samples, enzyme-labeled antibodies, and color development while maintaining fixed antigen coating and antibody concentrations.

#### 2.7.4 Model selection

Linear, double log-linear, 3-parameter probit, and 4-parameter logistic models were evaluated for precision, accuracy, and misjudgment probability (MMJP) to select the optimal model for data analysis using BMV V1.0 (NIFDC, China).

#### 2.7.5 Interference study of mucosal matrix

To minimize matrix interference, 15 nasal swab samples from COVID-19 convalescent individuals and 15 SARS-CoV-2 IgA-negative nasal samples were tested at starting sample-to-loading ratios of 1:2, 1:4, and 1:8. The ratio yielding the optimal specificity, sensitivity, and minimal matrix interference was selected.

### 2.8 Method validation

#### 2.8.1 Specificity

Specificity was assessed by testing 4-fold diluted nasal swab samples from 5 COVID-19 convalescent individuals, 20 pre-COVID-19 healthy individuals, 7 rhinovirus convalescent individuals, 4 influenza A virus convalescent individuals, 4 influenza B virus convalescent individuals, and 1 human metapneumovirus convalescent individual using the SARS-CoV-2 WT-RBD IgA detection method.

#### 2.8.2 Precision and accuracy

Per the Chinese Pharmacopoeia Part III (2020) (section 9401), precision and accuracy were evaluated by using repeated measurements of a national standard curve (3.125–100 U/mL), a total of 16 times, under two operators and at different times (30). Acceptance criteria: relative bias ≤ ± 20%, intermediate precision ≤ 20%.

#### 2.8.3 Method performance evaluation

BMV V1.0 software (NIFDC, China) was used to analyze method performance (SARS-CoV-2 WT and XBB.1.5 RBD mucosal IgA). Total analytical error, prediction intervals, tolerance intervals, capability indices, and misjudgment probability within the 3.125–100 U/mL range were evaluated.

### 2.9 SARS-CoV-2 WT-RBD IgA in NMLFs and serum of participants

The nasal samples collected by three sampling methods were detected using the ELISA method established in this study and the V-PLEX SARS-CoV-2 panel 33 (IgA) kit (Meso Scale Diagnostics (MSD), Rockville, MD 20850, USA) to determine the concentration of SARS-CoV-2 WT-RBD IgA. For ELISA, the concentration of coated WT-RBD IgA was 2 μg/mL, the concentration of the detection antibody W2 was 0.5 μg/mL, the incubation time for NMLFs samples was 60 min, and the color development time was 15 min. NMLF samples were diluted 1:4- or 1:400-fold. The National Standard for anti-SARS-CoV-2 mucosal IgA was diluted in a 2-fold dilution series, starting with an initial 10-fold dilution, to calculate the antibody concentrations (29). For the ECL assay, the NMLFs were diluted 1:90-fold. The operation was then based on the instructions of the V-PLEX SARS-CoV-2 panel 33 (IgA) kit. The ECL assay kit demonstrated a lower limit of quantification (LLOQ) of 0.14 AU/mL for WT RBD IgA (approximately 0.25 U/mL), with a dilution-adjusted LOQ of 22.5 U/mL. The National Standard for anti-SARS-CoV-2 mucosal IgA was diluted in a 4-fold dilution series, starting with an initial 4-fold dilution, to calculate the antibody concentrations. Serum samples diluted 1:5000-fold were detected in the V-PLEX SARS-CoV-2 panel 33 (IgA) kit.

### 2.10 Neutralization assay based on pseudoviruses

Pseudoviral neutralization assays against serum were carried out as previously described (31).

### 2.11 Statistical analyses

The statistical tests are indicated in the figure legends. Significance values are indicated by *p < 0.05, **p < 0.01, ***p < 0.001, and ****p < 0.0001; ns = insignificant. All statistical tests were performed using GraphPad Prism software.

To assess the level of agreement between ECL and novel ELISA established in this study, Lin’s concordance correlation coefficients were calculated using log WT-RBD IgA concentration for 146 clinical samples that have WT-RBD IgA concentration above the dilution-adjusted LOQ. Calculations were performed using the R package ‘DescTools.’ (32)

The robustness of the three sampling methods has been expressed using geometric coefficients of variation (GCV = (10^S^-1)×100%, where S is the standard deviation of the log concentration of total IgA or WT-RBD IgA) (33, 34).

Correlations between NMLFs and serum antibodies were tested by a two-sided Spearman correlation test using GraphPad Prism software.

## 3 Results

### 3.1 Characterization of immunoglobulin composition in serum and nasal mucosa

**Figure 1:**
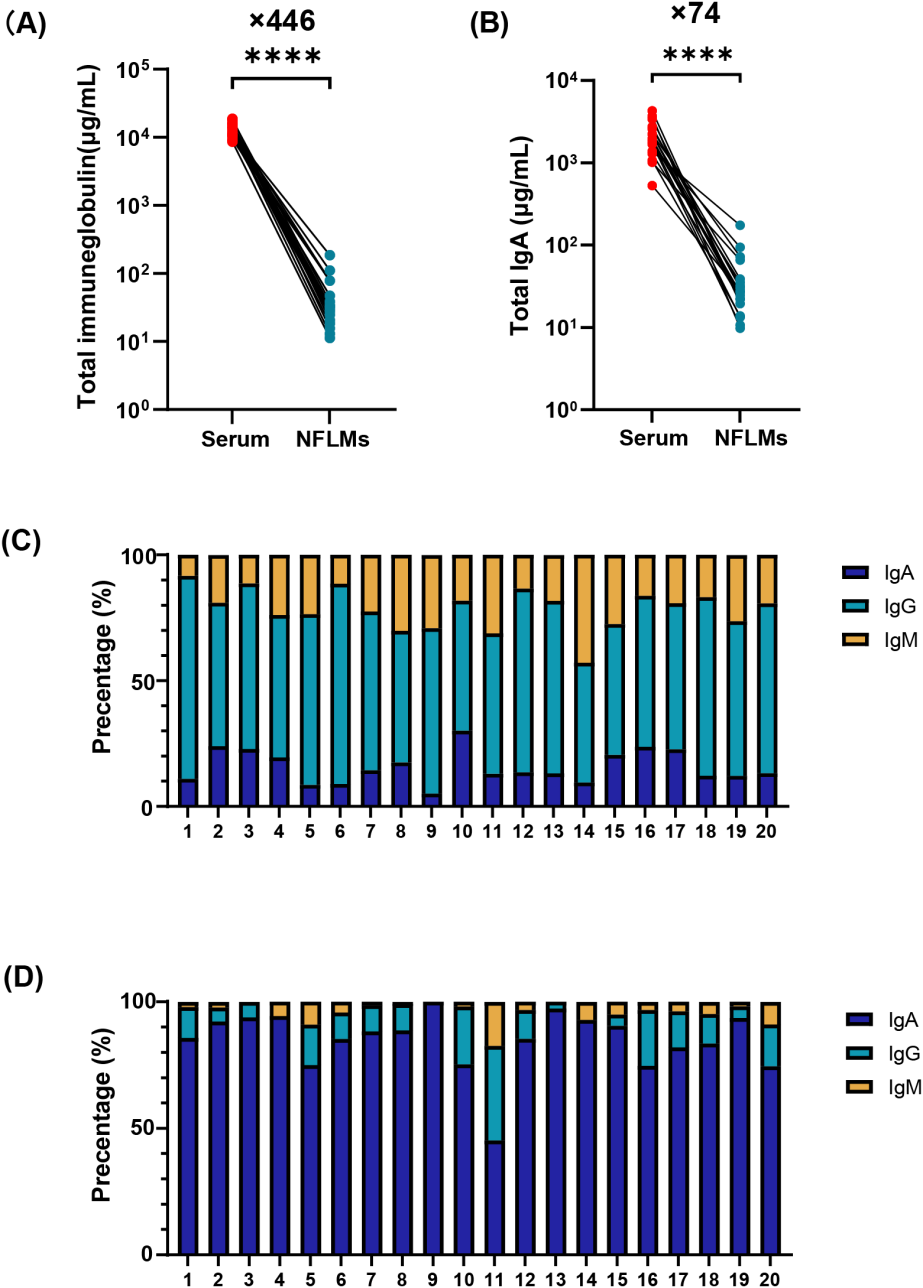
Immunoglobulin composition in serum and NMLFs from Group B numbered 1-20. Total immunoglobulin **(A)**, total IgA **(B)** concentrations in serum and nasal swabs were analyzed by paired t-test (**** p<0.0001). Proportions of IgA, IgG, and IgM antibodies in serum **(C)** and nasal swabs **(D)** using stacked bar charts. NMLFs, nasal mucosal lining fluids.

To characterize the immunoglobulin composition in the serum and nasal mucosa, paired serum and nasal swab samples from 20 COVID-19 convalescent individuals were analyzed using ECL. The results revealed significantly higher antibody levels in serum than in the nasal mucosa. Total immunoglobulins, IgA, IgG, and IgM in the serum were 446-fold (interquartile range, IQR: 210–596), 74-fold (IQR: 40–108), 2560-fold (IQR: 1093–3353), and 1825-fold (IQR: 968–3403) higher, respectively, than those in the nasal mucosa (**Figures 1A, B**, **Supplemenentary Figures S1B, C**). IgG was the dominant immunoglobulin in the serum, accounting for 62.3% (IQR: 56.5%–68.1%) of total immunoglobulins, with lower proportions of IgA and IgM (**Figure 1C**, **Supplemenentary Figure S1A**).

In contrast, nasal antibodies are mainly composed of IgA (**Figure 1D**, **Supplementary Figure S1A**). The median nasal IgA concentration was 25.6 μg/mL (IQR: 21.3–36.1 μg/mL), representing approximately 87.0% (IQR: 80.3%–93.0%) of total immunoglobulins. However, the proportions of IgG and IgM in some individuals were 37.3% and 17.5%, respectively (**Figure 1D**). Low antibody levels in the nasal mucosa pose significant challenges for the accurate and reliable quantification of mucosal IgA.

To address these challenges, efforts should focus on enhancing assay sensitivity and improving sample collection capabilities. This study optimized both detection and sampling methodologies to establish a standardized testing platform.

### 3.2 Establishment of a sensitive and specific nasal SARS-CoV-2 RBD IgA antibody detection method based on ICH Q14 & Q2(R2)

To guide the establishment and validation of analytical methods, the International Council for Harmonisation (ICH) issued the ICH Q14 and Q2(R2) guidelines. Q14 describes enhanced approaches that provide a systematic way to develop and refine knowledge of an analytical procedure, as well as demonstrate a thorough understanding of the procedure (35). Q2(R2) offers guidance on the selection and evaluation of analytical procedures (36). Developing and validating analytical methods in accordance with ICH Q14 and Q2(R2) ensures that the developed methods are fit for their intended purposes. Therefore, this study established the analytical method for detecting SARS-CoV-2 nasal IgA in compliance with ICH Q14 and Q2(R2).

#### 3.2.1 Method development

Based on the intended purpose and experimental experience, an ATP for the nasal SARS-CoV-2 RBD IgA antibody detection method was preliminarily established, specifying the requirements for specificity, precision, and accuracy (**Table 1**). Using a previously developed fishbone diagram of ELISA-influencing factors in our laboratory, potential factors affecting accuracy, precision, and specificity were prioritized, and a Failure Mode and Effects Analysis (FMEA) table was constructed (37). Factors with a risk priority score >60 were identified as critical risks. Optimal parameters were determined using fixed-factor screening and customized experiments (**Supplementary Table S2**).

**Figure 2:**
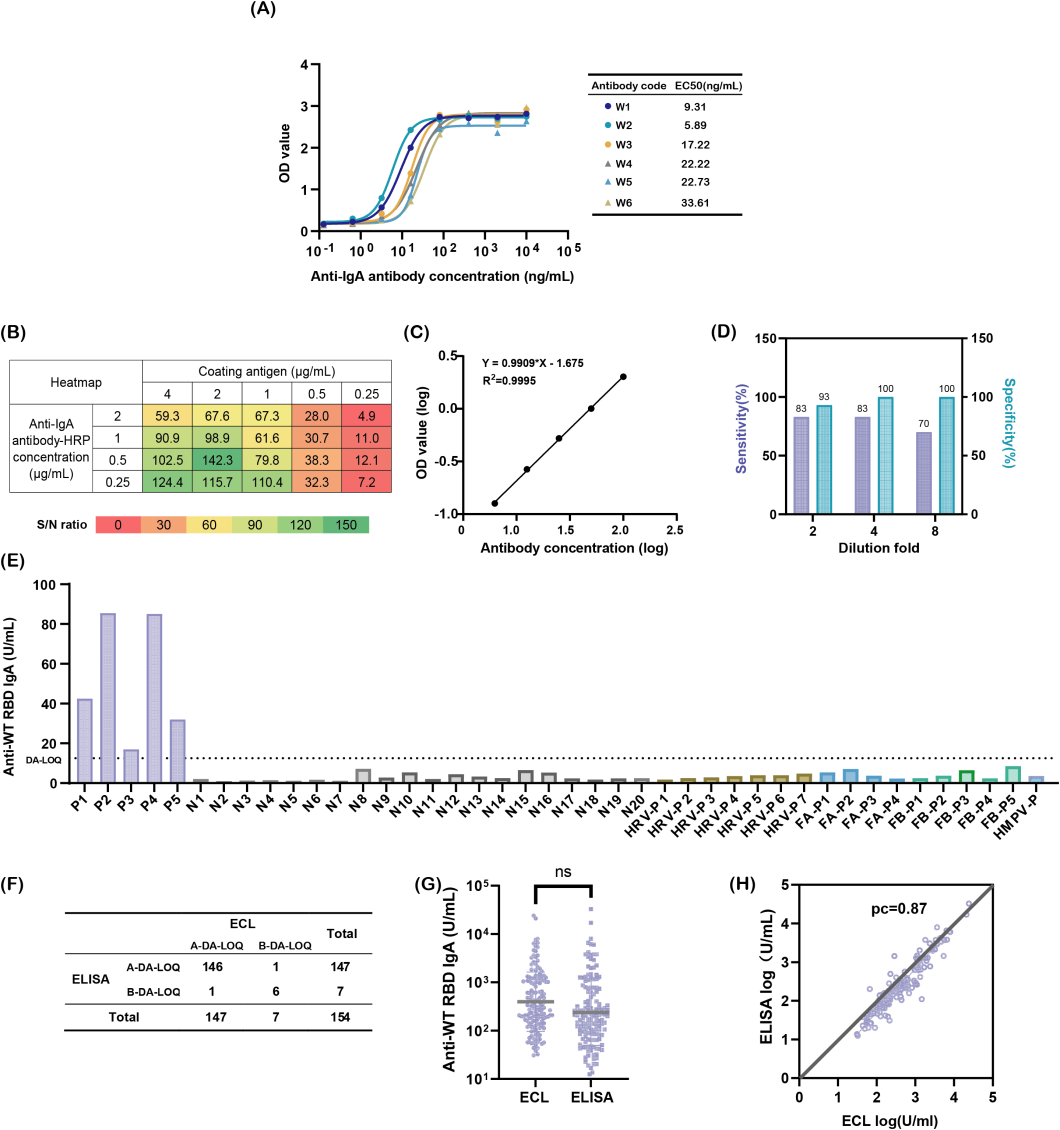
Developing a sensitive and specific method for detecting nasal SARS-CoV-2 WT-RBD IgA antibodies. **(A)** Binding affinity of six candidate antibodies to IgA; **(B)** Heatmap screening for optimal antigen coating concentrations and enzyme-labeled secondary antibody concentrations; **(C)** Reportable range of the assay: logarithmic plot of diluted National Standard for anti-SARS-CoV-2 mucosal IgA concentrations (x-axis) versus absorbance values (y-axis). *Note: Linearity was validated across sixteen replicates; one representative result is shown;* **(D)** Optimization of mucosal sample dilution ratios to minimize matrix interference; **(E)** Specificity validation: P (COVID-19-positive samples), N (pre-COVID-19 nasal swabs), HRV (rhinoviruses), FA (influenza A virus), FB (influenza B virus), HMPV (human metapneumovirus), dilution-adjusted LOQ (DA-LOQ); **(F)** Concordance rate between two detection methods, above the dilution-adjusted LOQ (A-DA-LOQ), below the dilution-adjusted LOQ (B-DA-LOQ); **(G)** Comparison of SARS-CoV-2 WT-RBD IgA antibody concentrations in 146 A-DA-LOQ mucosal samples measured using ECL and ELISA, analyzed using a paired *t*-test (ns: p > 0.05); **(H)** Concordance correlation coefficients for log results of ECL and ELISA.

Secondary antibody detection was identified as a critical factor influencing the specificity and sensitivity, prompting its optimization. Three commercial anti-IgA antibodies were evaluated first. However, none exhibited IgA-specific recognition. Commercial antibodies 2 and 3 cross-reacted with 3.1 μg/mL IgM, while commercial antibody 1 showed strong binding to 3.1 μg/mL IgG (**Supplementary Figure S2**). To meet the ATP criteria, six monoclonal antibodies with high IgA affinity were screened using indirect ELISA. Among these, the in-house antibody W2 demonstrated superior performance, with an EC_50_ of 5.89 ng/mL, which is significantly lower than that of the others, and exclusive specificity for IgA (**Figure 2A**). W2 exhibited robust binding to 12.5 μg/mL IgA, comparable to commercial antibodies 1 and 2 (**Supplementary Figure S2**), and was thus selected for further optimization.

Checkerboard titration was used to optimize the concentrations of coating antigen and enzyme-labeled secondary antibody. The highest signal-to-noise ratio (S/N = 142.3) was achieved at a 2 μg/mL antigen coating and a 0.5 μg/mL dilution of the enzyme-labeled secondary antibody (**Figure 2B**), which were subsequently used in the experiments.

Regarding the test sample incubation time, enzyme-labeled antibody incubation time, and color development time, a custom experiment was designed using the JMP 17 software (**Supplementary Table S3**), and simulations were conducted using Space Profiler. The results demonstrated that when the sample incubation time and enzyme-labeled antibody incubation time were both within 55–120 min, and the color development time was 12–15 min, the probability of achieving an S/N ratio greater than 20 in the detection results was 100% (**Supplementary Figure S3**). Consequently, these conditions were established as the parameter acceptance range (PAR) of the methodological influencing factors. For efficiency, the sample incubation time and enzyme-labeled antibody incubation time were set at 60 min, and the color development time was set at 15 min.

After optimization, the data were fitted to linear, double log-linear (x-logarithmic and y-logarithmic), 3-parameter probit, and 4-parameter logistic models. The double log-linear model (3.125–100 U/mL range) demonstrated an intermediate precision of <7%, relative bias of <7%, and misjudgment probability (MMJP) <1% across all concentrations, outperforming the other models (**Supplementary Tables S4**-**S6**).

#### 3.2.2 Method validation

The developed method exhibited excellent linearity (R² >0.99) within 3.125–100 U/mL (**Figure 2D**). The bias between the predicted and actual concentrations was <4%, with an intermediate precision of <10% for all concentrations except 3.125 U/mL (<17%) (**Supplementary Table S7**). BMV software analysis confirmed that prediction and tolerance intervals met specifications (90–112%) across the range, with method capability indices ≥3, the method misjudgment probability was <1%, and total analytical error <5% (**Supplementary Table S8**).

To address matrix interference from mucosal mucoproteins and lysozymes, the sample dilution was optimized. A 4-fold dilution balanced the specificity (93%) and sensitivity (70%), outperforming the 2-fold (lower specificity) and 8-fold (lower sensitivity) dilutions, respectively (**Figure 2D**).

Specificity was validated using 20 pre-COVID-19 mucosal samples, 5 COVID-19 convalescent samples, and 17 samples from patients infected with common respiratory viruses, including rhinovirus, influenza A/B, and human metapneumovirus. Only the COVID-19 convalescent samples exceeded the dilution-adjusted LOQ (12.5 U/mL), confirming no cross-reactivity (**Figure 2E**). The lower limit of quantification was validated to be 3.125 U/mL. The method meets the ATP criteria for accuracy, precision, and specificity.

To assess the versatility of the W2 antibody, the method was adapted for detecting XBB.1.5 RBD. Linearity (3.125–100 U/mL) was retained, with a bias of <7% and an intermediate precision of <19% (**Supplementary Figure S4**, **Supplementary Table S9**). Within 71–141% ranges, method capability indices ≥2 and total error ≤3% were achieved (**Supplementary Table S10**), demonstrating broad applicability for mucosal antibody quantification in other respiratory pathogens.

#### 3.2.3 Clinical validation of the SARS-CoV-2 WT-RBD IgA ELISA method

To compare the performance of the newly developed SARS-CoV-2 WT-RBD IgA ELISA method with ECL, nasal samples collected on day 1 from 154 participants across five groups using the expanded sponge method (M3) were detected by two methods. Among these, 146 samples exceeded the dilution-adjusted LOQ in both assays, 6 fell below the dilution-adjusted LOQ, and 1 sample exceeded the dilution-adjusted LOQ only in ELISA or ECL (**Figure 2F**). Using the ECL results as a reference, the ELISA method demonstrated 99.3% agreement for samples above the dilution-adjusted LOQ, 85.7% agreement below the dilution-adjusted LOQ, and a Kappa value of 0.85. For the 146 samples above the dilution-adjusted LOQ, ELISA results ranged from 12.5–32,828.9 U/mL (median: 200.83 U/mL), while ECL results ranged from 30.8–24,075.0 U/mL (median: 312.55 U/mL), with no significant difference (p = 0.0504) (**Figure 2G**). The concordance correlation coefficient (CCC) between the two methods was 0.87 (**Figure 2H**), indicating strong agreement [CCC >0.8, considered acceptable (32)]. These results confirm that the ELISA method developed under the ICH Q14 & Q2(R2) guidelines performs comparably to ECL and meets the clinical quantification requirements.

### 3.3 Comparative study of sampling methods

**Figure 3:**
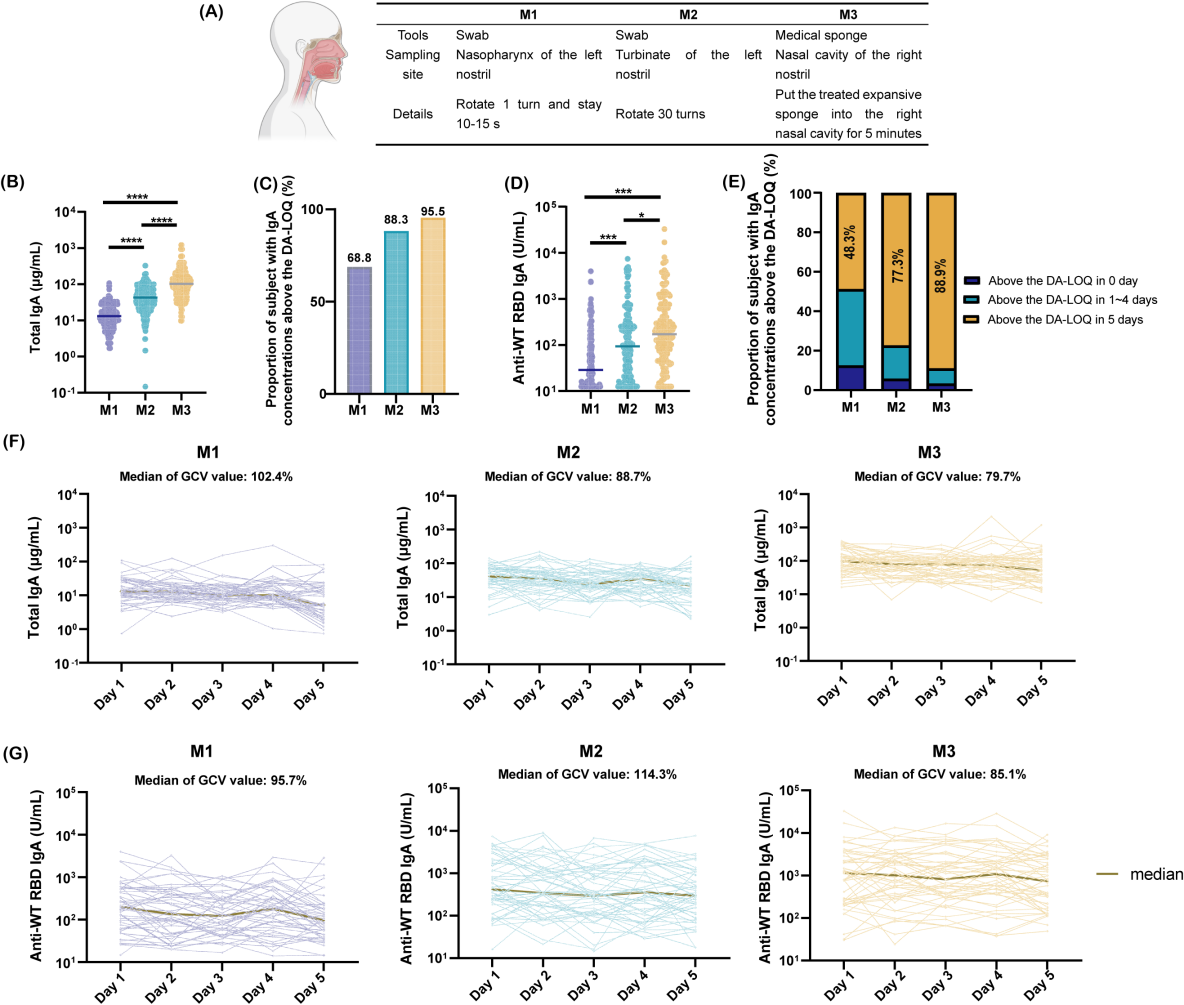
Comparison of nasal sampling methods. **(A)** Tools, collection sites, and procedural details for three sampling methods; **(B)** Total IgA concentrations from day 1 collections using three methods, analyzed by paired t-test (****p <0.0001); **(C)** Proportion of samples with SARS-CoV-2 WT-RBD IgA concentrations above the dilution-adjusted LOQ (DA-LOQ) for each method; **(D)** SARS-CoV-2 WT-RBD IgA concentrations from day 1 collections, analyzed using a paired *t*-test (*p<0.05; ***p <0.001); **(E)** Proportion of samples with SARS-CoV-2 WT-RBD IgA concentrations above the dilution-adjusted LOQ (DA-LOQ) over five consecutive days of sampling; **(F)** Total IgA concentrations from five consecutive days of sampling using M1, M2, and M3 (data shown only for 48 participants with consistent SARS-CoV-2 WT-RBD IgA levels above the dilution-adjusted LOQ (DA-LOQ); **(G)** WT-RBD IgA concentrations from five consecutive days of sampling (same subset as F).

To ensure accurate, robust, and comparable quantification of mucosal antibodies, we designed a clinical trial to conduct a comparative evaluation of clinical sampling methods. Through a literature review, we identified nasal lavage, nasal swabs, nasopharyngeal swabs, and absorbent matrix samples as the commonly used specimens for nasal testing. Nasal lavage was excluded from the study owing to its low acceptability and challenges in ensuring sample recovery rates (18). Nasopharyngeal swabs (M1), nasal swabs (M2), and the expanded sponge method (M3) were selected as study subjects because of their standardized procedures, high feasibility, and relatively high acceptability. The three sampling methods differed in terms of the tools, sampling sites, and specific operational protocols (**Figure 3A**).

From September to November 2023, 154 participants were recruited (**Supplementary Table S1**). 35 participants underwent a single collection (M1/M2 in the left nostril and M3 in the right nostril). A total of 119 participants underwent sampling for five consecutive days using all three methods.

NMLFs were analyzed by a human/NHP kit and the SARS-CoV-2 WT-RBD IgA ELISA method developed in this study. Results showed that M3 yielded a median total IgA concentration of 95.0 μg/mL (IQR: 51.6–151.7 μg/mL), which was 6.9- and 2.2-fold higher than those of M1 and M2, respectively (**Figure 3B**). For WT-RBD IgA, M3 achieved a detection rate of 95.5% above the dilution-adjusted LOQ, outperforming M1 (1.4-fold) and M2 (1.1-fold) (**Figure 3C**). The median WT-RBD IgA concentration for M3 was 171.2 U/mL (IQR: 64.6–627.5 U/mL), which was significantly higher than that for M1 and M2 (**Figure 3D**). Normalization of WT-RBD IgA to total IgA eliminated the variability of collection methods (**Supplementary Figure S5A**).

M3 demonstrated superior robustness with 88.9% of the samples remaining above the dilution-adjusted LOQ over five consecutive days, compared to 48.3% for M1 and 77.3% for M2 (**Figure 3E**). Among the 48 participants with consistent WT-RBD IgA levels above the dilution-adjusted LOQ, M3 exhibited lower geometric coefficients of variation (GCV) for total IgA (79.7%) and WT-RBD IgA (85.1%) than M1 and M2 (**Figures 3F, G**). Normalization further reduced the GCVs to 44.4% (M1), 30.4% (M2), and 39.8% (M3) (**Supplementary Figure S5B**).

In conclusion, the expanded sponge method (M3) offers enhanced sampling capability, higher antibody recovery, and improved robustness, making it a practical choice for nasal antibody quantification.

### 3.4 Application of the detection platform

**Figure 4:**
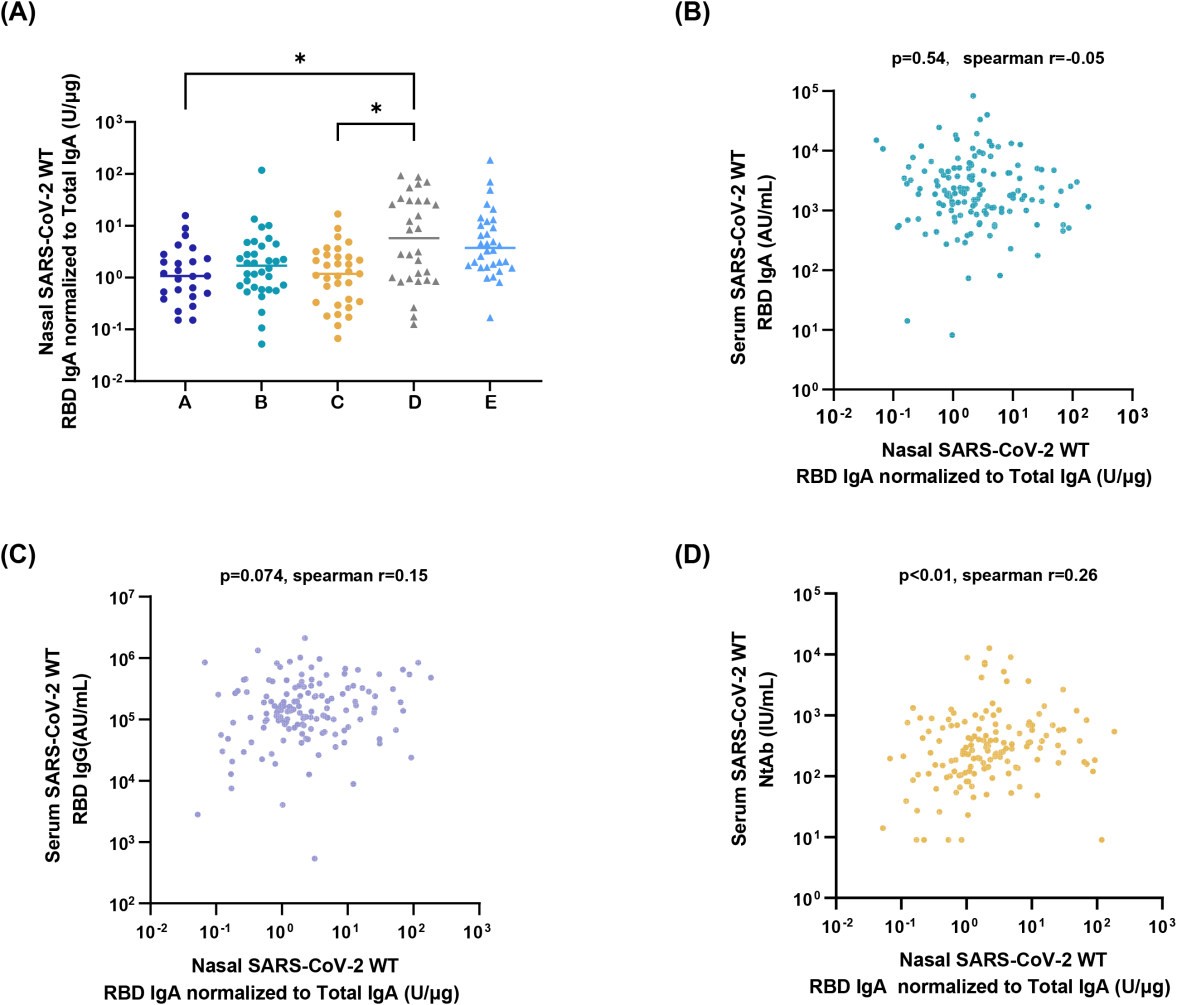
Application of the detection platform. **(A)** Normalized nasal SARS-CoV-2 WT-RBD IgA levels in samples collected on day 1 from the five groups using the M3 method. The data were analyzed using ordinary one-way ANOVA (*p < 0.05). Correlation analysis between normalized nasal SARS-CoV-2 WT-RBD IgA and serum IgA **(B)**, IgG **(C)**, and neutralizing antibodies **(D)**.

Utilizing the developed sensitive ELISA method, the high-capability sampling technique, and the first national standard for SARS-CoV-2 mucosal IgA, nasal SARS-CoV-2 WT-RBD IgA profiles were characterized across diverse populations. In the nasal samples collected on day 1 from groups A-E using the M3 method, the proportion of samples with SARS-CoV-2 WT-RBD IgA concentrations exceeding the dilution-adjusted LOQ was 88.5%, 93.9%, 97.0%, 93.3%, and 100%, respectively. (**Supplementary Figure S6A**). The live-attenuated influenza virus vector-based intranasal SARS-CoV-2 vaccine group (D) exhibited a median SARS-CoV-2 WT-RBD IgA concentration of 378.2 U/mL (IQR: 78.0–1845.2 U/mL), which was 5.1-, 2.6-, 2.4-, and 1.5-fold higher than A, B, C, and E, respectively (**Supplementary Figure S6B**). Normalization of SARS-CoV-2 WT-RBD IgA to total IgA revealed significantly higher specific antibody levels in group D than in groups A and C (**Figure 4A**). Notably, most individuals without COVID-19 symptoms (Group A) tested positive for SARS-CoV-2 WT RBD IgA, suggesting a widespread subclinical infection.

Serum samples were analyzed for SARS-CoV-2 WT-RBD IgA/IgG (via ECL) and neutralizing antibodies (via a pseudovirus neutralization assay). Normalized nasal SARS-CoV-2 WT-RBD IgA levels showed a weak correlation with serum neutralizing antibodies (r=0.26, p<0.01), but no correlation with IgA(r=-0.05, p>0.05), IgG (r=0.15, p>0.05) (**Figures 4B–D**). This highlights the inadequacy of serum antibody measurements in reflecting mucosal immunity and underscores the need for the detection of mucosal antigen-specific IgA.

## 4 Discussion

The respiratory mucosa serves as the primary line of defense against respiratory pathogens, and mucosal antibodies and immune cells play critical roles in the prevention of infections. Numerous studies have shown that levels of antigen-specific IgA are inversely correlated with the infection rates of pathogens, such as SARS-CoV-2 (13, 14, 38, 39). Thus, mucosal IgA is a key indicator of respiratory mucosal vaccine efficacy. To ensure the accurate quantification of nasal IgA, this study established a sensitive and specific ELISA method for SARS-CoV-2 IgA detection, coupled with a robust and efficient sampling technique, to form a standard clinical detection platform. Using this platform, we analyzed the nasal SARS-CoV-2 WT-RBD IgA in individuals with different infection statuses and in those who have received the SARS-CoV-2 mucosal vaccine. These results demonstrate that participants who received a single dose of a live-attenuated influenza virus vector-based intranasal SARS-CoV-2 vaccine have higher nasal SARS-CoV-2 WT-RBD IgA concentrations. Notably, only serum neutralizing antibody levels showed a weak correlation with nasal IgA (r = 0.26, p < 0.01), underscoring the need for direct assessment of nasal IgA rather than relying solely on serum biomarkers.

Adults produce 5–8 g of mucosal IgA daily, distributed across approximately 400 m² of mucosal surface, with substantial variation in IgA concentrations across different mucosal sites (39). Previous studies have shown that pulmonary antibody concentrations are approximately 200–500 folds lower than those in the serum (40). In this study, paired human nasal mucosa and serum samples were analyzed, revealing that the total immunoglobulin and IgA levels in the nasal mucosa were significantly lower than those in the serum, with fold differences of 446 and 74, respectively (**Figures 1A, B**). Subtype analysis of the nasal immunoglobulins identified IgA as the dominant immunoglobulin, accompanied by minor amounts of IgG and IgM (**Figure 1D**). The proportions of IgA and IgG were consistent with the findings of Kirkeby et al., who analyzed nasal lavage samples from 15 individuals (41). However, the proportion of IgM in certain subjects reached 17.5%, which exceeded the 3% reported in Kirkeby’s study. Elevated IgM levels may correlate with the recent health status of these individuals (42). Given the heterogeneity in antibody levels and health conditions among clinical study participants, establishing highly sensitive and IgA-specific mucosal antibody detection methods is critical to ensure the accurate quantification of mucosal IgA.

To address regulatory requirements for reliability, robustness, and accessibility, this study aligned with ICH Q14 and Q2(R2) guidelines to develop an ELISA method optimized for nasal samples (35, 36, 43). Through risk assessment and Design of Experiments (DoE), critical factors were prioritized, leading to the identification of W2, a monoclonal antibody with superior IgA specificity and sensitivity compared to commercial reagents. The optimized method demonstrated specificity, accuracy (relative bias <4%), intermediate precision <17%, and total error <5%, meeting ICH M10 criteria (44). The W2 antibody also enabled the successful adaptation of SARS-CoV-2 XBB.1.5 RBD IgA detection. This finding also indicates that by changing the coating antigen, this platform can be used to detect mucosal-specific IgA against other respiratory pathogens based on W2. Comparative analysis with ECL using 154 mucosal samples revealed high agreement between the methods (99.3% for samples above the dilution-adjusted LOQ, Kappa = 0.85) and strong quantitative concordance (CCC = 0.87), confirming the equivalence of ELISA and ECL for clinical use (**Figure 2**).

Nasal sample collection methods are diverse. Swabs, absorbent matrices, and nasal irrigators are commonly used sampling tools, and the nasopharynx and nasal turbinate are typical sampling sites. Studies have shown that absorbent matrices exhibit approximately five-fold higher collection capacity than nasal and nasopharyngeal swabs (26, 45). In this study, we compared the expanded sponge method (functionally analogous to absorbent matrices) with two swab-based methods and found that it enhanced the antibody collection capacity by up to 6.9-fold. In addition, we systematically evaluated the robustness of the three sampling methods by calculating the SARS-CoV-2 WT-RBD IgA detection rates over five consecutive days and the variability in total IgA and SARS-CoV-2 WT-RBD IgA concentrations across the same period. The expanded sponge method demonstrated superior performance in term of sustained detection rates and lower variability in both total and SARS-CoV-2 WT RBD IgA levels compared to the other two methods. Enhanced M3 sampling capability may correlate with a longer sampling time, a greater nasal cover area, and an appropriately selected sampling site. Research by the Crotty team revealed significant differences in immune cell populations between two adjacent upper respiratory regions: the nasopharynx harbors higher proportions of germinal center B cells, germinal center T follicular helper cells, and IgG+ B cells, whereas the nasal turbinate is enriched in antibody-secreting cells, IgA+ B cells, IgM+ B cells, memory B cells, and tissue-resident memory T cells (46). These findings suggest that nasal turbinate samples are suitable for evaluating specific mucosal immune responses induced by mucosal vaccines or respiratory pathogens, providing an additional rationale for selecting an expanded sponge method for turbinate sampling. This highlights the potential for future cell-mediated immune studies of the nasal mucosa using the expanded sponge method, as the sponge can reach the turbinate. Normalization of SARS-CoV-2 WT-RBD IgA using total IgA further reduced the variability in serially collected samples, which is consistent with the study of de Silva et al. (26).

In a phase II clinical study of a live attenuated influenza virus vector-based intranasal SARS-CoV-2 vaccine, researchers used an in-house ELISA method to measure SARS-CoV-2 WT-RBD IgA titers in nasopharyngeal swabs. They found that the positivity rate and geometric mean titer (GMT) of WT-RBD IgA in vaccine recipients were 12% and 3.8, respectively, slightly higher than those in the control group (2%, 3.5) (20). Based on our newly established nasal IgA antibody detection platform, this study revealed that 93.3% of individuals who received the live attenuated influenza virus vector-based intranasal SARS-CoV-2 vaccine tested above the dilution-adjusted LOQ three months post-vaccination, with significantly higher nasal SARS-CoV-2 WT-RBD IgA concentrations compared to asymptomatic individuals and convalescent individuals 10 months post-breakthrough infection. This discrepancy may be attributed to the enhanced sampling efficiency and sensitivity of our detection platform. For the adenovirus vector vaccine group, 100% of participants exhibited nasal IgA levels above the dilution-adjusted LOQ. However, the concentrations of SARS-CoV-2 WT-RBD IgA were not significantly higher than in other groups, aligning with previously reported findings (21). This may relate to the oral inhalation immunization route.

Using our established nasal antibody detection platform, nasal SARS-CoV-2 WT-RBD IgA levels across populations with varying infection statuses were further analyzed. Among individuals without COVID-19 symptoms (Group A), the nasal SARS-CoV-2 WT-RBD IgA positivity rate was 88.4%, consistent with predictions of subclinical infection rates in China by Fu et al. (47), suggesting the utility of our method as a robust tool for clinical epidemiological studies. The high baseline IgA levels in asymptomatic individuals also resulted in no significant differences in nasal SARS-CoV-2 WT-RBD IgA concentrations compared to those of convalescent individuals at 3 or 10 months post-infection.

Analyzing correlations between nasal and serum antibodies may aid in identifying surrogate biomarkers and elucidating the origin of mucosal antibodies. Our findings demonstrated only a weak correlation between nasal SARS-CoV-2 WT-RBD IgA and serum neutralizing antibodies, consistent with prior studies (14), underscoring the necessity of direct mucosal IgA assessment. Integrating findings from Crotty and Shulman’s teams (46, 48), we propose that nasal IgA antibodies are not derived from serum but are locally secreted by antibody-secreting B cells that migrate to the nasal turbinate.

This study has certain limitations. Firstly, the detection method has been applied for a short period, and sufficient longitudinal monitoring data remains to assess its performance. Subsequent efforts will focus on continuously monitoring methodological performance metrics to ensure ongoing compliance with the ATP. Secondly, the clinical investigation was designed as a cross-sectional study, which precludes the acquisition of longitudinal changes in individual serum and mucosal antibody levels or SARS-CoV-2 infection rates. Consequently, it cannot provide supporting data for determining the mucosal protective correlates.

## 5 Conclusion

Guided by ICH Q14 and Q2(R2), this study established the first standardized ELISA for nasal SARS-CoV-2 WT-RBD IgA detection, which was validated against ECL with >85% dilution-adjusted LOQ concordance and strong quantitative agreement (CCC = 0.87). The expanded sponge sampling method (M3) enhanced the percentage of the above dilution-adjusted LOQ (1.1–1.4-fold daily, 1.2–1.8-fold over 5 days) and robustness. Integrating this platform with the National Standard for anti-SARS-CoV-2 mucosal IgA (No. 300052-202401, 1000 U/mL) provides a reliable and scalable framework for evaluating mucosal vaccines, setting a precedent for standardizing mucosal antibody assays for other infectious diseases. With appropriate modifications, the standardized detection system established in this study can be adapted for the clinical evaluation of other respiratory mucosal vaccines, thereby advancing the development of mucosal vaccines.

## Data Availability

The original contributions presented in the study are included in the article/**Supplementary Material**. Further inquiries can be directed to the corresponding authors.
