# Supplementary material for: Exploring the standardized detection and sampling methods of human nasal SARS-CoV-2 RBD IgA
Source: Front Immunol. 2025 May 20;16:1571418. doi: 10.3389/fimmu.2025.1571418 (PMC12129971; doi:10.3389/fimmu.2025.1571418)
Supplement: Supplementary file 1 [file DataSheet1.docx]

Supplementary Material

# Supplementary Figures and Tables

- 1. **Supplementary Figures**

##
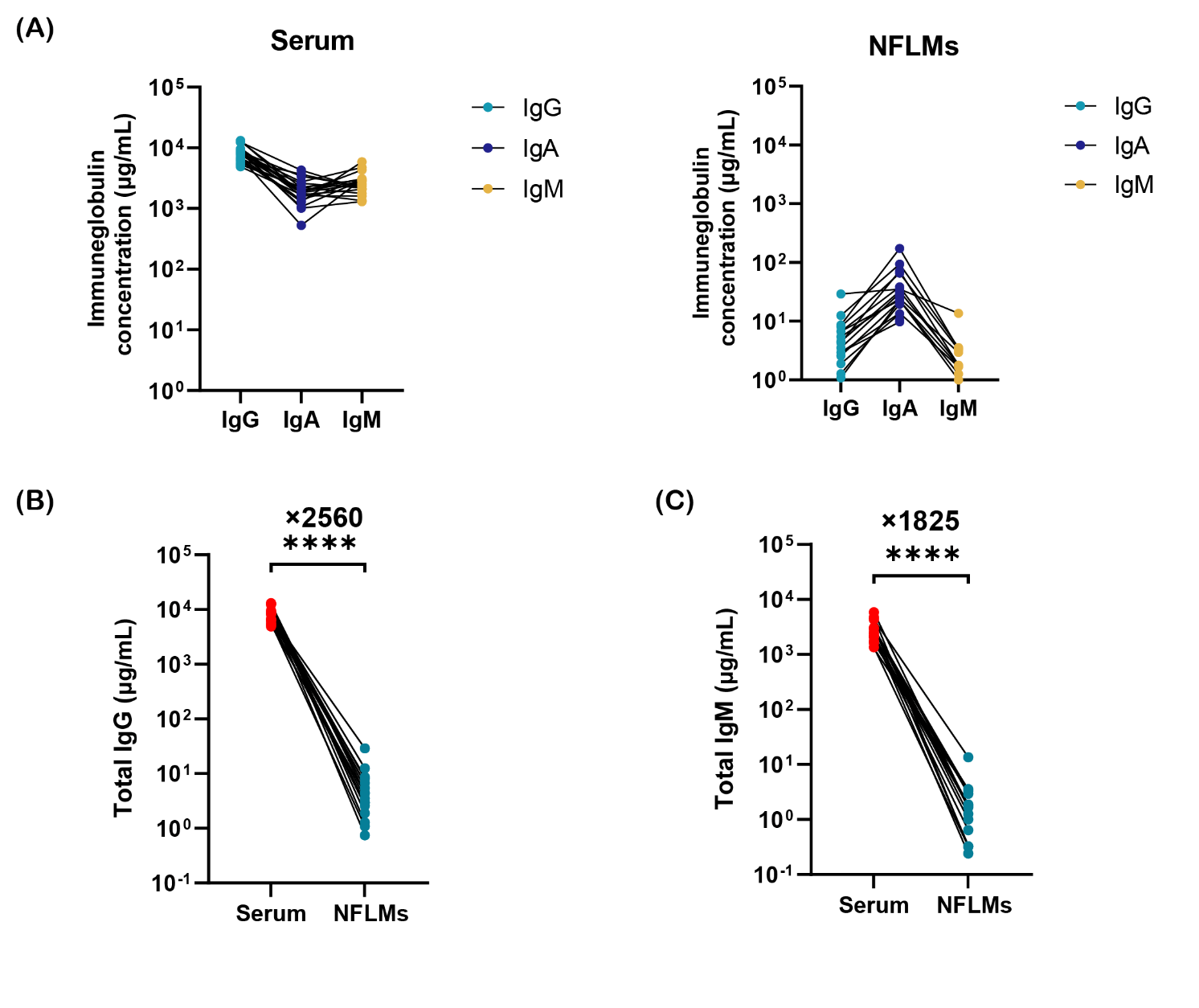


**Supplementary Figure 1.** Immunoglobulin composition in serum and NMLFs Group B numbered 1-20.. A: Serum IgA, IgG, and IgM concentrations; B: Total immunoglobulin concentrations in nasal swabs; C: Disparity in total IgG concentrations between serum and nasal swabs; D: Disparity in total IgM concentrations between serum and nasal swabs. A paired t-test was used to analyze the disparity in total IgG and IgM concentrations between the serum and nasal swabs (****, p < 0.0001). NMLFs: nasal mucosal lining fluids.


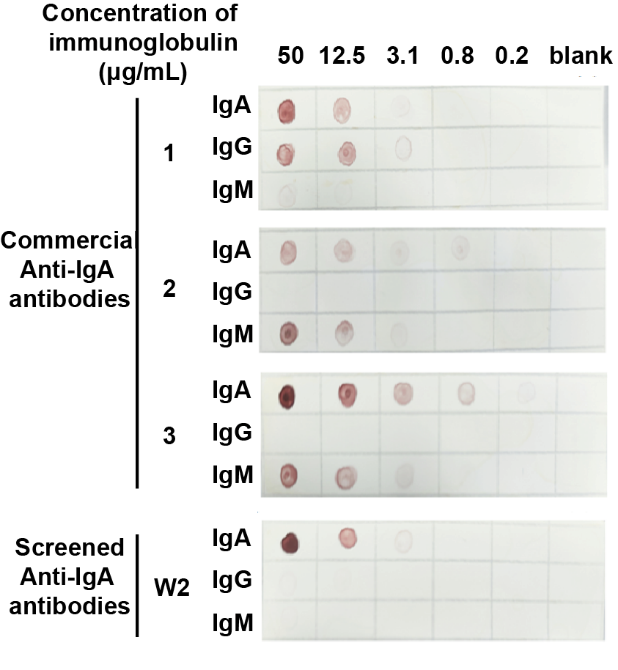


**Supplementary Figure 2.** Dot blot assay evaluating the binding capacity of three commercial anti-IgA antibodies and the in-house antibody (W2) to IgA, IgG, and IgM at varying concentrations (50, 12.5, 3.1, 0.8, 0.2 μg/mL).


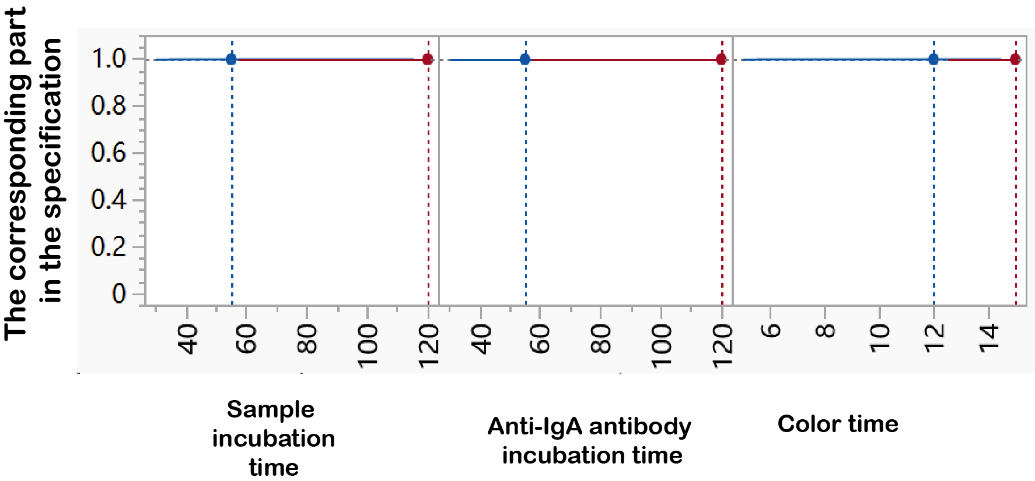


**Supplementary Figure 3.** Screening for optimal ELISA experimental conditions. The dynamics of each influencing factor (i.e., sample and anti-IgA antibody incubation time and color development time) were visualized using a space profiler. The red line indicates the corresponding part of the specification that varies with the upper limit. The blue line indicates the corresponding part of the specification that varies with the lower limit. The corresponding part of the specification close to 1 indicates a 100% test result if the signal-to-noise ratio value is greater than 4.


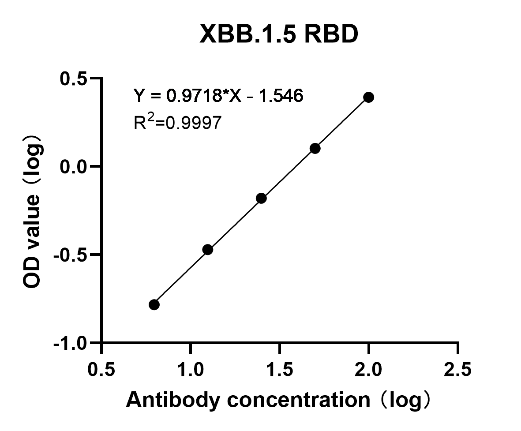


**Supplementary Figure 4.** Reportable range of the SARS-CoV-2 XBB.1.5 RBD IgA assay: logarithmic plot of diluted SARS-CoV-2 mucosal antibody standard concentrations (x-axis) versus absorbance values (y-axis). *Note: Linearity was validated across sixteen replicates; one representative result is shown.*

*
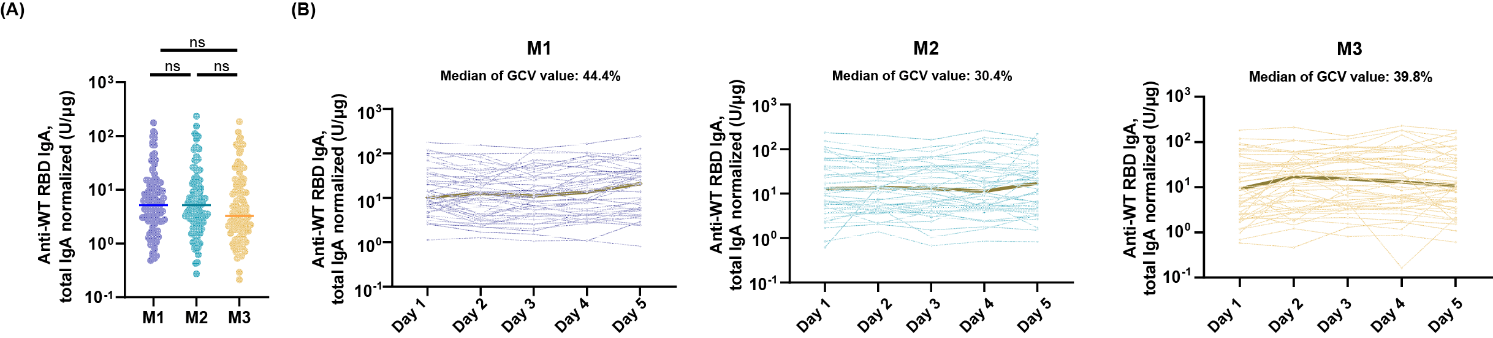
*

**Supplementary Figure 5.** Analysis of sampling efficiency based on the proportion of WT-RBD IgA to total IgA. **A:** Normalized nasal SARS-CoV-2 WT-RBD IgA in day 1 collections using M1, M2, and M3. Statistical analysis was performed using a paired t-test (ns, p >0.05). **B:** Normalized SARS-CoV-2 WT-RBD IgA in samples collected consecutively over five days using M1, M2, and M3. Only individuals with consistently positive collections across all five days are shown.

**
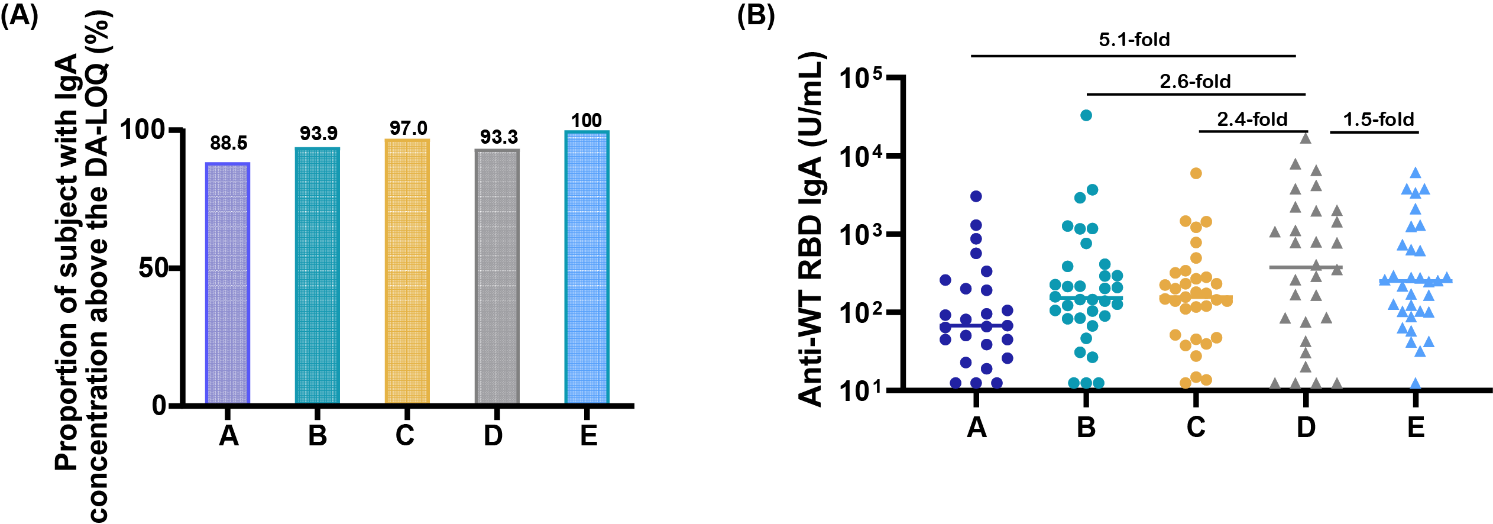
**

**Supplementary Figure 6.** Nasal SARS-CoV-2 WT-RBD IgA in day 1 collection using the M3 method. **A:** Detection rates of SARS-CoV-2 WT-RBD IgA above the dilution-adjusted LOQ (DA-LOQ) in each group. **B:** SARS-CoV-2 WT-RBD IgA levels in each group, analyzed using ordinary one-way ANOVA, with no significant differences observed between groups (thus, no markers are displayed).

- 1. **Supplementary Tables**

**Supplementary Table 1.** Demographics of the study cohort

|  | | **A(n=26)** | | **B(n=33)** | | **C(n=33)** | | **D(n=30)** | | **E(n=32)** |
| --- | --- | --- | --- | --- | --- | --- | --- | --- | --- | --- |
| **Sex** | |  | |  | |  | |  | |  |
| Female | | 17 (65%) | | 26 (79%) | | 31 (94%) | | 17 (57%) | | 20 (63%) |
| Male | | 9 (35%) | | 7 (21%) | | 2 (6%) | | 13 (43%) | | 12 (37%) |
| **Age** | |  | |  | |  | |  | |  |
| Median (Min, Max) | | 22（20, 53） | | 22（44,19） | | 21（19,53） | | 51（26,77） | | 50（22,73） |
|  | |  | |  | |  | |  | |  |
| **SARA-CoV-2 infection history** | | Without COVID-19 symptoms | | An interval of 0.5-3 months after the previous infection | | An interval of ≥10 months after the previous infection | | An interval of ≥10 months after the previous infection | | An interval of ≥10 months after the previous infection |
| **SARA-CoV-2 mucosal vaccine administration history** | | | | | | | | | | |
| Name (proportion, interval between vaccination time and sampling time for this study) | Ad5-nCoV_IH (19%, >1 year) | | Ad5-nCoV_IH (9%, >1 year) | | Ad5-nCoV_IH (3%, >1 year) | | LAIV_IN: (100%, ≤3 months) | | Ad5-nCoV_IH (100%, ≤3 months) | |
| **SARA-CoV-2 intramuscular vaccine administration history** |  | |  | |  | |  | |  | |
| One dose | | / | | / | | 1 (3%) | | / | | / |
| Multidoses | | 26 (100%) | | 33 (100%) | | 32 (97%) | | 30 (100%) | | 32 (100%) |
| Interval between the last vaccination time and sampling time for this study | | >1 year | | >1 year | | >1 year | | >1 year | | >1 year |

**Ad5-nCoV_IH**: adenovirus type 5 vectored SARS-CoV-2 vaccine through oral inhalation; **LAIV_IN**: Live-attenuated influenza virus vector-based SARS-CoV-2 vaccine through intranasal administration.

**Supplementary Table 2.** Failure mode effect analysis (FMEA) of ELISA method.

| Rating of importance |  |  | 8 | 8 | 10 |  |
| --- | --- | --- | --- | --- | --- | --- |
| ID | process step | process input | Accuracy | Specificity | Precision | Total |
| 1 | Environment | Temperature | 1 | 1 | 1 | 26 |
| 2 | Environment | Light | 1 | 1 | 1 | 26 |
| 3 | Environment | Humidness | 1 | 1 | 1 | 26 |
| 4 | Environment | Tidiness | 1 | 1 | 1 | 26 |
| 5 | Plate wash | Washing buffer | 1 | 1 | 1 | 26 |
| 6 | Microplate reader | Wavelength | 2 | 1 | 1 | 34 |
| 7 | Microplate reader | Brand | 2 | 1 | 1 | 34 |
| 8 | Microplate Washer | 96/12 holes | 2 | 1 | 1 | 34 |
| 9 | Calculation | Software | 2 | 1 | 1 | 34 |
| 10 | Blocking | Volume | 1 | 2 | 1 | 34 |
| 11 | Plate washing | Time | 1 | 1 | 2 | 36 |
| 12 | Test sample | Temperature | 1 | 1 | 2 | 36 |
| 13 | Enzyme secondary antibody | Temperature | 3 | 1 | 1 | 42 |
| 14 | Pipette | Electric/ manual | 2 | 1 | 2 | 44 |
| 15 | Coating buffer | Recipe | 1 | 2 | 2 | 44 |
| 16 | Analyst | Training | 2 | 1 | 2 | 44 |
| 17 | Analyst | Practice time | 2 | 1 | 2 | 44 |
| 18 | Analyst | Habit | 2 | 1 | 2 | 44 |
| 19 | Washing buffer | Recipe | 1 | 2 | 2 | 44 |
| 20 | Blocking buffer | Recipe | 1 | 2 | 2 | 44 |
| 21 | Test sample | Volume | 2 | 1 | 2 | 44 |
| 22 | Enzyme secondary antibody | Volume | 2 | 1 | 2 | 44 |
| 23 | Substrate | Volume | 2 | 1 | 2 | 44 |
| 24 | Enzyme labeling plate | Brand | 2 | 1 | 2 | 44 |
| 25 | Consumable | Absorbance | 2 | 1 | 2 | 44 |
| 26 | Coating | Time | 2 | 1 | 2 | 44 |
| 27 | Coating | Volume | 1 | 1 | 3 | 46 |
| 28 | Substrate | Brand | 2 | 2 | 2 | 52 |
| 29 | Sample dilution buffer | Recipe | 2 | 2 | 2 | 52 |
| 30 | Enzyme secondary antibody dilution buffer | Recipe | 2 | 2 | 2 | 52 |
| 31 | Substrate | Temperature | 2 | 2 | 2 | 52 |
| 32 | Washing plate | Volume | 2 | 2 | 2 | 52 |
| 33 | Microplate Washer | Maintenance | 2 | 2 | 2 | 52 |
| 34 | Blocking | Time | 2 | 2 | 2 | 52 |
| 35 | Blocking | Temperature | 2 | 2 | 2 | 52 |
| 36 | Incubator | Incubation mode | 2 | 1 | 3 | 54 |
| 37 | Coating | Temperature | 2 | 1 | 3 | 54 |
| 38 | Calculation | Model | 3 | 1 | 3 | 62 |
| 39 | Test sample | Concentration | 3 | 1 | 4 | 72 |
| 40 | Test sample | Time | 3 | 1 | 4 | 72 |
| 41 | Enzyme secondary antibody | Concentration | 3 | 1 | 4 | 72 |
| 42 | Enzyme secondary antibody | Time | 3 | 1 | 4 | 72 |
| 43 | Analyst | Ability | 4 | 1 | 4 | 80 |
| 44 | Coating | Concentration | 4 | 1 | 4 | 80 |
| 45 | Calculation | Concentration point setting | 4 | 1 | 4 | 80 |
| 46 | Enzyme secondary antibody | Brand | 3 | 4 | 4 | 96 |
| 47 | Substrate | Time | 4 | 3 | 5 | 106 |

Note: The primary evaluation metrics for this method are defined as accuracy, precision, and specificity, with weighting coefficients assigned as 8, 8, and 10, respectively. Each factor influencing these metrics is scored based on its potential impact level, where 1 represents the weakest influence and 5 the strongest. The total score for each influencing factor is calculated by multiplying its impact score for each primary metric by the corresponding weighting coefficient and summing the results.

Supplementary Table 3. Experimental schedule of the ELISA method targeting the WT RBD

| Test | Test sample incubation time (min) | Enzyme secondary antibody incubation time (min) | Color development time (min) | S/N | N |
| --- | --- | --- | --- | --- | --- |
| 1 | 120 | 75 | 10 | 266.8 | 0.0051 |
| 2 | 75 | 30 | 10 | 243.6 | 0.004 |
| 3 | 75 | 75 | 15 | 251.3 | 0.005 |
| 4 | 75 | 120 | 10 | 228.1 | 0.0041 |
| 5 | 120 | 120 | 15 | 570.1 | 0.0033 |
| 6 | 120 | 30 | 5 | 215.2 | 0.0035 |
| 7 | 30 | 120 | 15 | 139.0 | 0.0052 |
| 8 | 75 | 75 | 10 | 324.5 | 0.003 |
| 9 | 75 | 75 | 5 | 105.5 | 0.0059 |
| 10 | 30 | 30 | 15 | 172.7 | 0.0046 |
| 11 | 30 | 30 | 5 | 90.8 | 0.0043 |
| 12 | 120 | 30 | 15 | 357.6 | 0.0045 |
| 13 | 30 | 75 | 10 | 99.4 | 0.0063 |
| 14 | 120 | 120 | 5 | 298.3 | 0.0036 |
| 15 | 30 | 120 | 5 | 69.3 | 0.0052 |

S/N: signal-to-noise ratio; N: noise value.

Supplementary Table 4. Comparison of the intermediate precision of four models (the ELISA method targeting the WT RBD)

| Fitting model | Concentration（U/ml） | | | | | | |
| --- | --- | --- | --- | --- | --- | --- | --- |
|  | 3.125 | 6.25 | 12.5 | 25 | 50 | 100 | 200 |
| Linear | 2.48 | 1.38 | 3.19 | 5.13 | 5.47 | 13.89 | / |
| Double log-linear | 6.69 | 4.67 | 5.41 | 3.67 | 1.69 | 3.73 | / |
| 3 parameter probit | 9.70 | 4.32 | 5.15 | 4.47 | 3.34 | 8.19 | 5.78 |
| 4 parameter logistic | 99.37 | 7.48 | 6.28 | 4.54 | 3.08 | 7.85 | 7.07 |

/: not tested

Supplementary Table 5. Comparison of the relative accuracy of four models

(WT-RBD method)

| Fitting model | Concentration（U/ml） | | | | | | |
| --- | --- | --- | --- | --- | --- | --- | --- |
|  | 3.125 | 6.25 | 12.5 | 25 | 50 | 100 | 200 |
| Linear | 65.13 | 88.44 | 101.13 | 104.29 | 103.22 | 98.99 | / |
| Double log-linear | -5.20 | 1.10 | 4.92 | 3.77 | 0.53 | -4.67 | / |
| 3 parameter probit | 51.87 | 25.90 | 8.50 | -3.13 | -6.10 | 4.45 | -1.08 |
| 4 parameter logistic | -54.66 | 0.73 | 8.35 | 2.40 | -3.31 | 1.44 | -0.33 |

/: not tested

Supplementary Table 6. Comparison of the misjudgment probability of four models

(WT RBD ELISA method)

| Fitting model | Concentration（U/ml） | | | | | | | | | | | | | | |
| --- | --- | --- | --- | --- | --- | --- | --- | --- | --- | --- | --- | --- | --- | --- | --- |
|  | 3.125 | 6.25 | | 12.5 | | | 25 | 50 | | | 100 | | 200 | |  |
| Linear | 1 | | 1 | | 1 | 1 | | | 1 | 1 | | / | |  |  |
| Double log-linear | 4.03E-10 | | 0 | | 0 | 0 | | | 0 | 0 | | / | |  |  |
| 3 parameter probit | 9.50E-01 | | 1.97E-09 | | 0 | 0 | | | 0 | 2.22E-16 | | 0 | |  |  |
| 4 parameter logistic | 9.10E-01 | | 0 | | 0 | 0 | | | 0 | 0 | | 0 | |  |  |

/: not tested

**Supplementary Table 7.** Analysis of relative accuracy and intermediate precision

(WT-RBD ELISA method, n=16）

| Antibody concentration （U/mL） | 3.125 | 6.25 | 12.5 | 25 | 50 | 100 |
| --- | --- | --- | --- | --- | --- | --- |
| Relative accuracy (Bias, %） | 1.94 | 0.19 | -1.47 | -1.96 | -1.92 | 3.35 |
| Intermediate precision（%） | 16.05 | 8.87 | 8.00 | 6.86 | 6.53 | 5.78 |

**Supplementary Table 8.** Evaluation of method capability at different concentration levels

(WT-RBD ELISA method）

| Antibody concentration （U/mL） | Method variability（%） | 90% Tolerance interval（%） | 90% Prediction interval（%） | MCI | MMJP | Method level |
| --- | --- | --- | --- | --- | --- | --- |
| 100 | 1.35 | 95.48-111.86 | 96.97-110.14 | 1.03 | 8.96E-11 | Ⅲ |
| 50 | 1.70 | 93.26-103.15 | 94.19-102.14 | 1.63 | 1.78E-14 | Ⅱ |
| 25 | 1.75 | 93.10-103.24 | 94.04-102.20 | 1.58 | 1.52E-13 | Ⅱ |
| 12.5 | 1.97 | 94.18-103.08 | 95.01-102.18 | 1.81 | 1.44E-11 | I |
| 6.25 | 2.45 | 96.84-103.66 | 97.48-102.47 | 2.41 | 5.43E-13 | I |
| 3.125 | 4.97 | 94.75-109.67 | 96.11-108.11 | 1.12 | 1.74E-04 | Ⅲ |

MCI: Method Capability Indices; MMJP: Misjudgment Probability.

**Supplementary Table 9.** Analysis of relative accuracy and intermediate precision

(XBB.1.5-RBD ELISA method, n=16）

| Antibody concentration （U/ml） | 3.125 | 6.25 | 12.5 | 25 | 50 | 100 |
| --- | --- | --- | --- | --- | --- | --- |
| Relative accuracy (Bias, %） | -6.90 | 1.50 | 6.56 | 5.54 | -0.08 | -5.83 |
| Intermediate precision（%） | 18.46 | 14.18 | 9.88 | 5.56 | 4.26 | 7.73 |

**Supplementary Table 10.** Evaluation of method capability at different concentration levels

(XBB.1.5-RBD ELISA method）

| Antibody concentration （U/ml） | Method variability（%） | 90% Tolerance interval（%） | 90% Prediction interval（%） | MCI | MMJP | Method level |
| --- | --- | --- | --- | --- | --- | --- |
| 100 | 1.27 | 82.46~107.91 | 84.66~105.11 | 1.91 | 0 | I |
| 50 | 1.14 | 96.23~103.82 | 96.94~103.05 | 6.76 | 0 | I |
| 25 | 1.18 | 92.97~119.72 | 95.30~116.79 | 2.03 | 0 | I |
| 12.5 | 1.37 | 92.01~122.87 | 94.65~119.44 | 1.78 | 0 | I |
| 6.25 | 1.51 | 95.32~107.22 | 96.35~105.98 | 4.34 | 0 | I |
| 3.125 | 2.67 | 77.22~112.57 | 80.63~108.57 | 1.39 | 6.31E-09 | II |

MCI: Method Capability Indices; MMJP: Misjudgment Probability.
